# Determinants of fish consumption among older adults in the UK

**DOI:** 10.3389/fnut.2025.1716700

**Published:** 2025-11-28

**Authors:** Mandu Stephen Ekpenyong, Adetoro Ogunleye, Aishat T. Bakre

**Affiliations:** 1 School of Nursing and Public Health, Manchester Metropolitan University, Manchester, United Kingdom; 2 Accord UK Ltd., Barnstaple, United Kingdom; 3 Department for Health and Social Care, University of Wales Trinity Saint David, Carmarthen, United Kingdom

**Keywords:** fish consumption, older adults, dietary behaviour, barriers and enablers, qualitative research

## Abstract

**Background:**

Eating fish regularly can lower the risks of heart disease, brain disorders, and overall mortality, especially in older adults. Even with these benefits, older people tend to eat less fish than younger people, and the reasons behind these eating habits are not well understood.

**Aim:**

This study investigated the determinants of fish consumption among older adults in the UK. It focused on what they see as helpful or limiting factors and their beliefs about health.

**Methods:**

The study used a qualitative approach with two focus group discussions involving 12 participants aged 60 and older, recruited from a local religious organisation in Wolverhampton, UK. Data was collected through semi-structured interviews based on available literature and expert advice. The transcripts were analysed using thematic analysis to find key themes. Rigour was maintained through reflection, the use of different moderators, and repeated coding.

**Results:**

Participants explained that their fish consumption is influenced by cultural traditions, religious beliefs, family habits, and personal tastes. Helpful factors included taste, health beliefs, and convenience. Barriers included cost, difficulty in preparation, and availability. Many participants acknowledged the physical and mental health benefits of eating fish, but they also expressed concerns about quality, authenticity, and misconceptions regarding frozen fish. These results show how dietary choices are affected by a mix of personal, social, and environmental factors.

**Conclusion:**

Although older adults acknowledge the nutritional and health benefits of fish, practical barriers such as affordability, accessibility, and preparation challenges continue to limit intake. Addressing these barriers and promoting lifelong positive habits may support improved consumption and healthier ageing.

## Introduction

Fish consumption plays a significant role in global health by lowering the risk of illness and death (1). Fish is rich in essential nutrients such as vitamins, minerals, and amino acids (2–4). This makes it an important part of a balanced diet (5). Research suggests that eating more fish is linked to a lower risk of cardiovascular diseases (6), respiratory diseases (7), and some cancers (8, 41). Furthermore, nutritious contributions vary by fish type: oily species are high in long-chain omega-3 fatty acids (EPA and DHA) that support cardiovascular and neuro-metabolic health (9), while lean fish provide lower fat but higher protein density and elevated levels of micronutrients such as iodine and taurine (9, 10). When combined in the diet, lean and oily fish therefore enhance the breadth of nutrient delivery, which may be particularly relevant for older adults seeking to support muscle mass, metabolic health, and long-term function.

Fish is also a source of long-chain omega-3 polyunsaturated fatty acids, which support brain health (11, 12) and lower the risk of dementia (13). Regular fish consumption has been associated with improved quality of life and lower risk of mortality in later life (14, 15). However, evidence on whether older adults consume less fish than younger individuals remains equivocal. Some studies suggest a decline in fish intake with advancing age (13), whereas other investigations, such as the longitudinal Tromsø Study of older Norwegian adults, found higher intake of both lean and fatty fish associated with lower odds of pre-frailty (OR = 0.63 for fatty fish) at 8-year follow-up (16). The reasons for this lower fish consumption among older adults are not well explored, especially through qualitative methods that capture personal experiences (17, 18).

Quantitative research has found connections between fish consumption and factors such as age, sex, marital status, economic status, health conditions, costs, and access (13, 18, 19). However, these studies do not fully capture the complex experiences influencing dietary choices. The present study aimed to employ qualitative focus group discussions (FGDs) to explore barriers and facilitators of fish consumption among older adults in the United Kingdom, with particular attention to how nutritional considerations interact with attitudes, habits and socio-economic circumstances. Understanding these factors is crucial for creating effective strategies to encourage fish intake and improve health outcomes in this group.

## Materials and methods

### Study design

A qualitative design employing focus group discussions (FGDs) was adopted to explore older adults’ experiences with fish consumption. FGDs were preferred over individual interviews for their efficiency and ability to generate rich insights through group interaction (20, 21).

### Participants and recruitment

Participants were older adults aged 60 and older living in Wolverhampton, UK. Recruitment occurred through a local religious organisation, using email invitations and newsletter announcements. Twelve participants consented to join, with equal representation of genders. Most participants were White British (83.3%), with two identifying as Black British. Sample size determination was informed by the methodological principle of data saturation. The average age of participants was 67.6 years (SD ± 10.3), and each focus group had six participants. Focus group discussions were carried out on 21st September 2018, following ethical approval from the University of Wolverhampton Faculty Ethics Subcommittee. Participants were affiliated with a Christian church and a Muslim community in Wolverhampton, with the majority (*n* = 8) identifying as Pentecostal, (*n* = 2) as Catholic, and (*n* = 2) as Muslim. This composition reflects the religious and cultural diversity of the local community, which provided a valuable context for exploring how faith-based norms and social networks influence food consumption patterns.

The inclusion of participants from a predominantly Christian yet religiously mixed background allowed the research to capture a range of perspectives on dietary practices, while maintaining the cohesion needed for group dialogue. Recruiting within an existing faith community was methodologically justified because it fostered trust, familiarity, and open communication among participants, conditions particularly important when engaging older adults on personal lifestyle behaviours. The purposive sampling approach also aligns with qualitative research principles prioritising depth of insight over representativeness, ensuring a rich understanding of the social and cultural factors underpinning fish consumption in later life.

### Data collection

Semi-structured interview guides were developed based on existing literature and expert consultation (22–24) and reviewed by one subject expert in nutrition and behavioural sciences (acknowledged in the Acknowledgements section). The guide comprised open-ended questions, such as: What makes you choose or avoid eating fish? How do you usually buy or prepare fish?, and what challenges do you face when eating fish? Sociodemographic data were collected, including age, gender, education, monthly income (in Pound Sterling), religion, family, and decision-making in food purchasing. The average UK monthly income for older adults is approximately £1,400, with the poverty threshold at 60% of the national median income (about £1,100). These contextual figures were included to situate participants’ financial capacity relative to national benchmarks.

FGDs lasted for about 60 min and were moderated by ATB and two co-moderators (MES and AO). Discussions were audio-recorded, and field notes captured non-verbal communication and observations from the setting. Focus group discussion interviews were conducted in a private room of the church. Approximately three weeks were spent in the church environment by the researchers prior to data collection to build rapport and trust through informal interaction. The exploratory design of the interviews allowed for meaningful engagement with participants’ lived experiences.

### Data analysis

The audio recordings were transcribed verbatim and imported into NVivo v11. The transcripts were reviewed multiple times for familiarisation and initial coding. Using thematic analysis (25), codes were generated inductively and iteratively grouped into main themes and sub-themes. The analysis maintained rigour by comparing codes across the two groups, discussing differences, and critically assessing the researchers’ assumptions. Diverse and conflicting perspectives were intentionally explored to provide a detailed, context-sensitive understanding of older adults’ experiences with fish consumption. The analysis was reflective and critical, recognising that participants’ beliefs and actions are influenced by overlapping social, cultural, economic, and environmental factors. This method helped identify both facilitators and barriers, illustrating the complexity of dietary choices without oversimplifying the issue.

### Trustworthiness

The study’s trustworthiness was strengthened through a mix of methodological and analytical methods. For this study, credibility was achieved by conducting two focus groups with separate moderators, capturing various viewpoints, and cross-checking findings with field notes and repeated transcript evaluations. Dependability was supported by detailed documentation of the analytical process and independent reviews of themes by two researchers. Reflexivity was essential for confirmability, as the research team critically reflected on their assumptions and interpretations to minimise bias. Transferability was enhanced by describing participant characteristics, household practices, cultural influences, and environmental contexts in detail, allowing readers to judge the relevance of the findings to similar groups. By including participant stories and considering both aligned and contrasting viewpoints, the study offers a thorough understanding of the factors affecting fish consumption among older adults.

### Ethical considerations

Each prospective participant received a detailed information sheet before data collection, which clearly explained the aims, procedures, and voluntary nature of the study. Participants were explicitly reminded of their autonomy, including the right to withdraw at any stage without consequence. To minimise the risk of coercion, all questions were phrased in a non-directive manner. Informed consent was secured either through a signed consent form or, where preferred, recorded verbal consent, in alignment with institutional ethical protocols. Additionally, the researcher maintained a reflexive journal as a means of critically engaging with issues of positionality and potential bias throughout the research process.

Ethical approval was obtained from the Health Professions, Psychology, Social Work, and Social Care ethics subcommittee of the Faculty of Education, Health and Wellbeing at the University of Wolverhampton in July 2018.

## Results

Table 1 shows the demographic characteristics of the research participants.

**Table 1:** Demographic characteristics of the research participants (*n* = 12).

Demographic characteristics	*N* (%) of participants
Age (yrs) mean ± SD	67.6 years ±10.3
Sex	
Males	8 (66.7)
Female	4 (33.3)
Marital status	
Married	6 (50.0)
Never married	1 (8.3)
Widow	5 (41.7)
Income (£)	
800–1,000	4 (33.3)
1,000–3,000	6 (50.0)
>3,000	2 (16.7)
Occupational class	
Public sector	2 (16.7)
Private sector	1 (8.3)
Self-employed	1 (8.3)
Retired	8 (66.7)
Educational background	
Secondary school	3 (25.0)
University degree	3 (25.0)
Graduate	3 (25.0)
Other	3 (25.0)
Ethnicity	
White British	10 (83.3)
Black British	2 (16.7)
Religion	
Pentecostal	8 (66.7)
Catholic	2 (16.7)
Muslim	2 (16.7)
Family	
Yes	9 (75.0)
No	3 (25.0)

**Figure 1:**
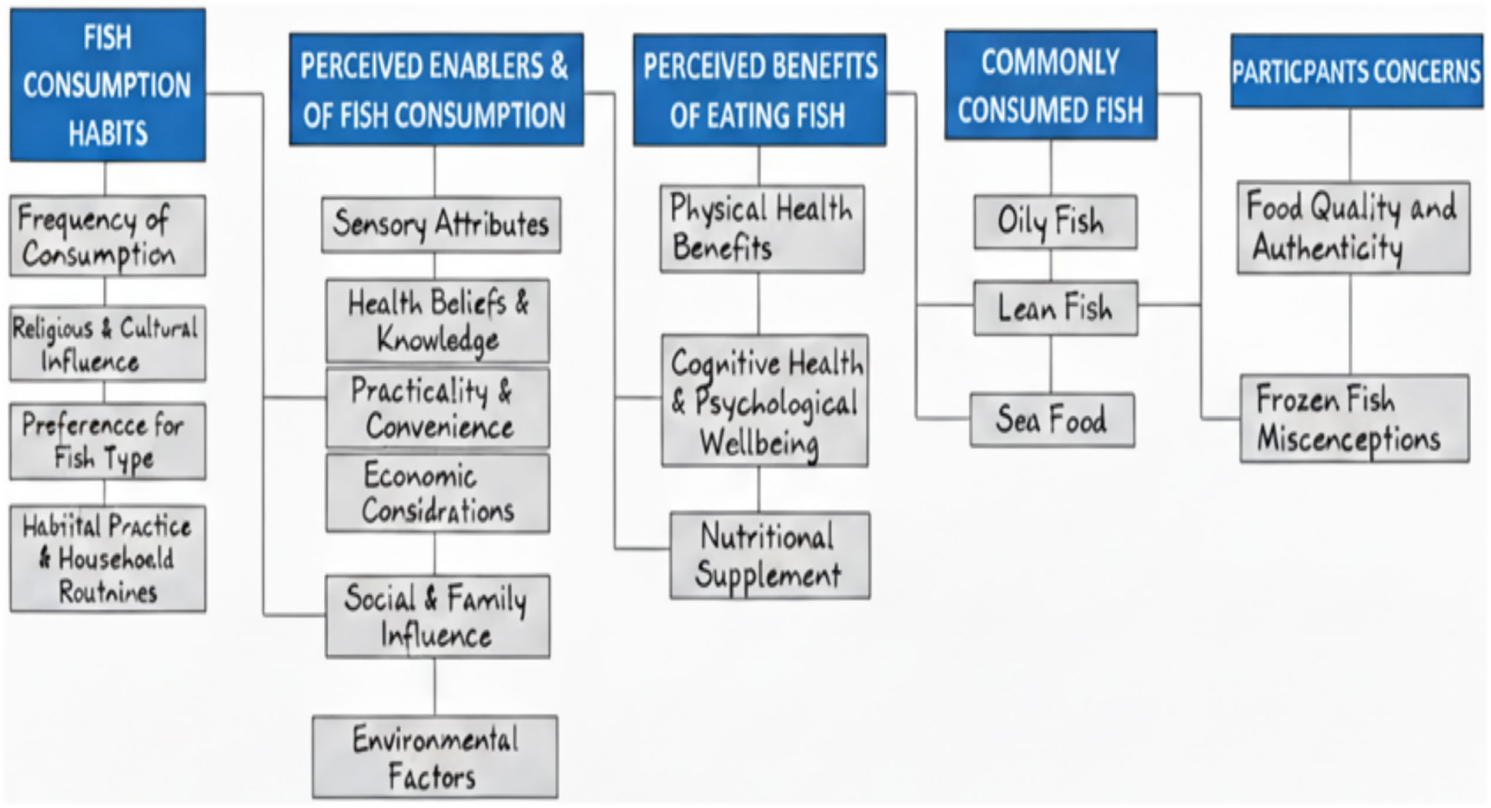
Main themes and sub-themes on determinants of fish consumption among older adults in the United Kingdom.

Five overarching themes emerged from the data collected during the two focus group discussions. These were fish consumption habits, perceived enablers/barriers to fish consumption, perceived benefits of eating fish, commonly consumed fish, and participants’ concerns. See Figure 1 for the schematic representation of the main themes and sub-themes in fish consumption. The role of this schematic representation of the main themes and sub-themes is to show how they all function to influence fish consumption among the study participants.

### Theme 1: Fish consumption habits

Patterns and routines around fish consumption were shaped by personal preferences, cultural and religious practices, and family habits. As shown in Figure 1, fish consumption habits among participants included frequency of consumption, religious and cultural influences, preferences for types of fish, habitual practices, and household routines.

#### Frequency of consumption

Participants reported eating fish anywhere from rarely (less than once per month) to weekly (typically one to two times per week), depending on individual habit and availability. Consumption patterns were frequently inconsistent and depended on household routines.

*“I am really a vegetarian. I do very occasionally eat fish. I rarely eat it.”* (P5 FG1)

*“I love fish, but I do not have a set menu. It can be once a month; it can be once every two months.”* (P6 FG1)

*“For me, it’s usually once or twice a week, depending on what’s available at home.”* (P2 FG2)

#### Religious and cultural influence

Religious and cultural traditions strongly influenced when participants ate fish, creating lifelong habits and promoting regular consumption on specific days.

*“…we always eat fish on Friday. So, it stays with you.”* (P5 FG2)

*“In my family, fish is part of our diet for religious reasons; we never skip it on Fridays.”* (P3 FG2)

*“It’s a habit from childhood; my parents insisted on fish at least once a week.”* (P1 FG2)

#### Preference for fish type

Choices between oily and lean fish depended on taste, texture, and perceived health benefits, reflecting individual dietary styles and health considerations.

*“I love big fish like mackerel; that is my oily fish.”* (P5 FG2)

*“I prefer salmon; it’s easy to cook and tastes good.”* (P4 FG1)

*“I mostly eat cod because it’s less oily and easier on my teeth.”* (P6 FG2)

#### Habitual practices and household routines

Family practices, cooking routines, and the availability of fish at home affected how often participants included it in meals.

*“…there are different ways of eating fish…at least once or possibly twice a week.”* (P5 FG2)

*“We usually keep tinned fish in the cupboard, so it’s convenient for meals.”* (P2 FG2)

*“If my spouse cooks fish, we’ll eat it more often; otherwise, it’s rare.”* (P4 FG2)

### Theme 2: Perceived enablers and barriers to fish consumption

This theme highlights the various factors that either encourage or impede fish consumption, such as sensory experiences, health beliefs, practical challenges, social and family influences, economic factors, and environmental accessibility.

#### Sensory attributes

Taste, smell, and texture were key influences on whether fish was eaten regularly or avoided.

*“The taste is good. That is what we know for sure.”* (P6 FG1)

*“…like you mentioned, it’s the flavour that matters most to me.”* (P2 FG1)

*“I sometimes skip oily fish because the smell lingers in the kitchen.”* (P2 FG2)

#### Health beliefs and knowledge

Understanding the health benefits of fish motivated participants to include it more often in their diets.

*“…if you know it is good for you, you are likely to buy more and include it in your diet. Fish is very healthy, especially because it has omega-3.”* (P3 FG2)

*“I eat fish because it’s said to be good for memory and heart health.”* (P1 FG2)

*“I try to include fish weekly; I believe it helps prevent diseases like heart problems and memory loss.”* (P5 FG1)

#### Practicality and convenience

Ease of preparation and the availability of frozen or ready-made fish influenced participants’ ability to consistently incorporate fish into their meals.

*“…some fish are very complicated to prepare…so I avoid them.”* (P4 FG2)

*“Frozen fish is convenient and saves cooking time.”* (P3 FG2)

*“I often buy ready-prepared fillets to avoid dealing with bones.”* (P6 FG2)

#### Economic considerations

Cost and household income impacted how often fish was eaten and the types that could be chosen, with some participants cutting back when prices were high.

*“Cost can be a barrier. If you do not have the money, you cannot afford to buy it as often as you want.”* (P3 FG2)

*“…fish can be quite pricey at times, especially salmon and tuna.”* (P1 FG2)

*“I adjust my consumption based on what I can afford each week.”* (P4 FG1)

*“I have never had an income issue that bothered me. My philosophy is that if I want to eat something I like, I do not check my wallet. I’d rather buy fewer clothes but the best fish.”* (P6 FG1)

*“I think income matters a lot, especially since many might prefer oily fish, and those can be quite expensive, especially salmon, tuna, and fresh tuna.”* (P2 FG2)

#### Social and family influence

Household makeup, spousal preferences, and the presence of children affected fish consumption patterns and meal planning.

*“…with young children around, family fish consumption might increase.”* (P4 FG1)

*“My spouse’s dislike of fish limits how often we eat it.”* (P4 FG2)

“*I eat more fish when I host family meals.”* (P1 FG2)

#### Environmental factors

Proximity to markets, living near the coast, and transport availability affected access to fresh fish and shaped consumption habits. Living in the Mediterranean was another factor that influenced fish consumption among the participants. Two mentioned that being born and living most of their lives in coastal areas affected how much fish they consumed.

*“I can eat quite a bit of fish, as it is key to the Mediterranean diet…if you have a fishmonger close by, you tend to go there often.”* (P3 FG2)

*“Having grown up in Galway and lived there for the last 20 years, I’ve noticed how the city’s coastal location influences people’s relationship with seafood. When mackerel come close to shore, many locals go fishing.”* (P1 FG2)

*“It’s well-known that people living near water, such as lakes or seas, tend to be healthier due to their fish-rich diets. For instance, the Inuit, who traditionally have few dietary options and rely heavily on raw fish, still maintain good health.”* (P2 FG2)

*“Living near the coast makes fresh fish easier to find.”* (P5 FG2)

“*Transport issues mean I often rely on tinned or frozen fish.”* (P6 FG1)

### Theme 3: Perceived benefits of eating fish

Fish was seen as beneficial for physical, cognitive, and nutritional health, reinforcing motivation to include it in the diet.

#### Physical Health benefits

Participants believed fish helped with heart health, weight management, and overall well-being.

“…blood pressure and cholesterol… all those things improve with eating fish.” (P3 FG2)

“*Fish is lighter than meat and easier to digest, so I prefer it for my health.”* (P4 FG2)

*“Eating fish helps me maintain my weight and energy.”* (P6 FG1)

#### Cognitive health and psychological well-being

Fish was associated with improved memory, mood, and overall mental health.

*“…my husband feels good and happy when he eats fish.”* (P4 FG1)

*“I feel mentally sharper when I eat fish regularly.”* (P1 FG2)

“*Fish makes me feel satisfied and less stressed about meals.”* (P5 FG2)

#### Nutritional supplementation

Participants recognised fish as a natural source of essential nutrients that support general health.

*“…fish contains iron and helps the body strengthen, including the brain, the eyes, and even the bones.”* (P4 FG2)

*“It’s a natural supplement; I do not need extra pills if I eat fish.”* (P3 FG2)

*“I include oily fish because of omega-3; it feels like medicine in food.”* (P6 FG1)

*“Fish is considered beneficial for people of all ages, particularly the elderly. One reason is that it contains relatively little fat. When older adults visit a doctor or hospital, their weight is often among the first health indicators assessed. Physicians frequently advise patients to manage their weight, which typically involves reducing the intake of fatty foods. Fish may therefore represent a healthy dietary option, as it is both low in fat and easily digested. In addition, its soft texture makes it suitable for individuals who have trouble chewing. Furthermore, regular fish consumption has been associated with supporting brain health.”* (P4 FG2)

### Theme 4: Commonly consumed fish

Choices of fish reflected a combination of preference, practicality and perceived health benefits.

#### Oily fish

Salmon, mackerel, tuna and kippers were preferred for taste, health benefits, and versatility.

*“I suppose the main mackerel and salmon…mainly oily fish.”* (P1 FG2)

*“Salmon is my favourite; I eat it at least once a week.”* (P3 FG2)

*“Tuna is convenient and healthy; I include it often.”* (P2 FG2)

#### Lean fish

Cod, haddock, and plaice were selected for texture, ease of preparation and digestibility.

*“I do cod and haddock mainly, easy to cook.”* (P3 FG2)

*“Plaice is simple and soft for chewing.”* (P6 FG1)

*“Lean fish fits my diet; I eat it more than red meat.”* (P4 FG2)

#### Seafood

Participants primarily consumed prawns, mussels and crab on special occasions or during family meals.

*“We like prawns and mussels, usually with pasta or family meals.”* (P3 FG2)

“*Seafood is occasional; I eat it when hosting family.”* (P1 FG2)

*“Crab is a treat; not part of daily routine.”* (P5 FG1)

### Theme 5: Participants’ concerns

Concerns about quality, authenticity and frozen products influenced confidence and willingness to consume fish.

#### Food quality and authenticity

Participants were concerned about production methods, additives and potential mislabelling.

*“…sometimes they are not really fish. How do we identify what we eat that we call fish?”* (P6 FG1)

*“It’s hard to know if fish is safe; sometimes labels are misleading.”* (P3 FG2)

*“I worry about chemicals in farmed fish; I prefer trusted sources.”* (P4 FG2)

#### Frozen fish misconceptions

Frozen fish was sometimes seen as inferior despite its convenience and nutritional value.

*“People often hold the misconception that frozen foods are not fresh, but frozen fish can be the next best thing. In fact, the cod we purchase frozen almost looks as if it has just come from the sea. In many cases, frozen fish may even be fresher than the fish sold by the fishmonger.”* (P3 FG2)

*“I often avoid frozen fish thinking it’s inferior.”* (P6 FG1)

*“Frozen fish is convenient, but I still prefer fresh whenever possible.”* (P2 FG2)

## Discussion

This study explored the perceptions and views of older adults on the determining factors that affect fish consumption. We identified five overarching themes: fish consumption habits, perceived enablers/barriers, perceived benefits, commonly consumed fish, and participants’ concerns.

The habitual fish consumption patterns reported by participants in this study reflect culturally embedded routines and generational practices. Many older adults described eating fish on specific days or favouring particular species due to lifelong habits and taste familiarity. Such patterns are consistent with evidence that older persons maintain established food preferences and are less inclined to vary their diet (26). While this continuity may support stable nutrient intake, it can also restrict dietary variety when preferred fish become less accessible or affordable.

In terms of fish consumption habits, respondents cited familiarity, ease of preparation, and legacy preferences (for example, salmon or cod) as key drivers. However, consumption frequency often fell short of optimal guidance, signalling possible nutritional vulnerability. This aligns with findings from the Tromsø Study, where a higher frequency of lean and fatty fish intake in older adults was associated with a lower odds of pre-frailty (OR ~0.69 for highest vs. lowest intake) (16). Participants voiced concerns about fish quality, environmental sustainability, and trust in supply chains. These concerns mirror the systematic review findings that cost, sensory or physical barriers, and health/nutritional beliefs (including concern over contaminants) are prominent deterrents to seafood consumption (26). Furthermore, cohort data from older adults show that regular fish consumers had lower all-cause mortality in those free of dementia (27), suggesting that overcoming access and trust barriers may yield substantial health benefits.

Environmental and contextual constraints were also evident. Some participants reported living in urban settings with limited fish variety, or facing mobility challenges for shopping for fresh fish, while others with coastal proximity highlighted easier access and stronger cultural traditions tied to fishing communities. These findings support the argument that behavioural interventions must consider not only individual motivators but also structural influences, availability, cost, preparation ease, and food environment.

Taken together, these findings underline that fish consumption in older adults is shaped by a complex interplay of lifetime habits, nutrition-driven health motivations, and barriers related to cost, sensory perceptions, and environment. Interventions aiming to increase fish intake in this group should therefore be multifaceted, addressing practical access (for example, frozen or tinned fish options), sensory and preparation support (simple recipes, deboned fish), and improving trust and knowledge about fish products.

The current study reveals income as an influential factor that can both facilitate and constrain fish consumption among older adults. This aligns with the findings of Can, Günlü, and Can (28), who identified income as the most significant determinant influencing household fish consumption, and with Thong and Søgaard (29), who reported that limited household income posed a substantial barrier to purchasing fish and shrimp. Similarly, Barberger-Gateau et al. (30), in a large-scale study of 9,280 French adults aged 65 years and above, observed that higher income levels were associated with more frequent fish consumption.

In contrast, a Nigerian cross-sectional study found that participants with higher income levels spent proportionally less on fish, indicating reduced consumption (31). This inverse relationship highlights the cultural dimension of dietary status, whereby fish, particularly local or freshwater varieties, is often perceived as a low-status food, associated with subsistence diets rather than affluence. In many African and some Asian contexts, red meat and poultry symbolise wealth, modernity, and social prestige, whereas fish may represent tradition or economic limitation. Consequently, the decline in fish consumption with increasing income in such settings may reflect not simply dietary preference but a status-driven shift in food identity. Recognising these socio-cultural associations provides a more nuanced understanding of how economic capacity interacts with cultural symbolism to shape dietary behaviour.

### Comparative discussion with studies in adult populations

The fish consumption habits reported in the current study correspond with patterns previously described among general adult populations. Taste, health considerations, and convenience were identified as the main drivers of seafood consumption in mixed-age samples across Australia, Belgium, and Scandinavia (22, 23). Our participants’ emphasis on taste and ease of preparation echoes these broader findings, suggesting that such core motivators remain consistent throughout the life course.

Consistent with earlier research (19, 24), our results also demonstrate that cost, access, and sensory attributes (for example, smell, bones) continue to constrain fish intake. These similarities suggest that the fundamental determinants of fish consumption, pleasure, health, convenience, and affordability, are pervasive, yet they manifest differently with advancing age. While much of the adult-focused literature provides a valuable foundation, it often predates 2015 and rarely disaggregates data by age. Where analyses do exist, older cohorts consistently report lower fish consumption, particularly those on fixed incomes or living alone (32). This underlines the importance of treating older adults as a distinct demographic, shaped by different social, physical, and nutritional contexts that influence dietary behaviour.

### Comparative discussion with studies in older-adult populations

Recent studies specifically examining fish consumption among older adults reinforce the significance of our findings. For example, a large Japanese cohort found that high fish consumption was associated with markedly reduced all-cause mortality in participants with elevated C-reactive protein (HR 0.49, 95% CI 0.26–0.92), suggesting an anti-inflammatory survival benefit (33). Similarly, longitudinal work in Korean older adults showed that fish and total seafood consumption were inversely associated with the prevalence of frailty (OR 0.47 and OR 0.34, respectively) at four years (34). In Norwegian older adults, a pattern of consistently high fish intake over 21 years was associated with 41% lower odds of pre-frailty (OR 0.59, 95% CI 0.38–0.91) (16).

Our findings, in which participants linked fish intake to perceived improvements in heart health, memory, and mood, align with these epidemiological results and reinforce the plausibility that habitual fish consumption contributes to healthier ageing trajectories. Nevertheless, practical and economic barriers, particularly affordability, accessibility, and preparation difficulties, may obstruct older adults from achieving the recommended intake levels necessary to realise these benefits.

Although the enablers identified here (taste, health, convenience) mirror those observed among younger groups, our data and contemporary literature highlight several age-specific determinants. Older adults may face physiological and contextual constraints, impaired dentition, sensory decline, reduced manual dexterity, or mobility limitations that hinder the preparation and consumption of fish (35). Additionally, fixed incomes or reliance on pensions heighten the salience of cost as a barrier compared with working-age adults (32).

Social context is also critical; living alone, bereavement, or reduced family mealtime structures diminish social facilitation effects known to encourage healthy eating. Conversely, faith-based traditions (for example, eating fish on Fridays) persist strongly among older cohorts, sustaining habitual consumption even when other motivators weaken. In addition, the participants’ perception in the current study showed that childhood/family with children can positively influence the consumption of fish is consistent with the findings of Thorsdottir et al. (36).

These distinctions support the argument that determinants of fish consumption in later life cannot be reduced to generic dietary drivers but must be understood through the lens of ageing physiology, income security, and social connectedness.

### Integration with nutritional and immunological implications

Fish is a principal source of long-chain omega-3 polyunsaturated fatty acids [PUFAs; Eicosapentaenoic acid (EPA), and Docosahexaenoic acid (DHA)] and high-quality protein, nutrients essential for maintaining immune competence, musculoskeletal function, and cognitive health in ageing. Systematic reviews show that omega-3 supplementation may exert small to moderate beneficial effects on muscle strength and function in older adults (37, 38). Moreover, fish-based intake has been associated with lower systemic inflammation. In the Japanese cohort, C-Reactive Protein (CRP) concentrations partially mediated the survival benefit of high fish consumption (33).

Our qualitative findings reinforce these nutritional implications; participants who recognised fish as “good for the memory and the heart” displayed an intuitive understanding of the biological mechanisms linking diet, inflammation, and cognitive health. In contrast, those deterred by preparation difficulty or misconceptions about frozen fish may risk suboptimal intake of these critical nutrients, potentially accelerating sarcopenia, frailty, or immune decline. This interplay between perception and physiology underscores the need to address practical barriers within interventions promoting fish consumption among older adults.

### Implications for dietary adequacy and ageing health outcomes

Each barrier or enabler identified in this study has tangible implications for dietary adequacy and health outcomes in ageing. For instance, economic barriers such as the higher cost of oily fish can lead to inadequate protein and omega-3 intake, contributing to sarcopenia and frailty (34, 39). Social enablers, including communal or family meals, can increase fish consumption frequency and thereby support better cognitive and cardiovascular health (15, 40). Convenience and confidence in preparation influence adherence to nutrient-rich diets. Educational initiatives promoting simple, affordable fish-based meals could therefore enhance dietary adequacy and extend healthspan.

Integrating these behavioural determinants within nutritional policy is essential. Interventions that promote affordable, pre-prepared, or locally sourced fish options may mitigate age-related nutritional deficiencies and indirectly support immune resilience, cognitive maintenance, and longevity.

### Strengths and limitations of the study

A notable strength of this study is its focus on an under-researched group, older adults, whose dietary behaviours and decision-making contexts differ substantially from those of younger populations. By using qualitative focus groups, the research provides rich, context-sensitive insight into how ageing, cultural norms, and economic constraints shape fish consumption. However, as a single-method qualitative study with a modest sample size drawn from one community, its findings are contextually specific and not intended for statistical generalisation. The depth of understanding achieved must therefore be interpreted within the limits of transferability rather than representativeness.

## Conclusion

This study provides insight into the complex and interrelated factors shaping fish consumption among older adults. Beyond general motivators such as taste, variety, and perceived health benefits, the findings emphasise the importance of convenience and physical capability in dietary decision-making during later life. Physiological changes associated with ageing, including reduced taste acuity, chewing, and limited dexterity, emerge as significant influences that affect both the preference for and the practicality of consuming fish. Economic and environmental considerations continue to play a role; however, barriers linked to ease of preparation, sensory comfort, and autonomy in cooking appear more decisive for this population. Interventions should therefore move beyond nutritional messaging to incorporate practical strategies, such as promoting deboned, pre-prepared, or easy-to-cook fish options that accommodate declining physical ability and sensory perception.

Furthermore, the findings suggest that older adults may perceive diminishing marginal health gains from dietary changes at advanced ages, which can reduce motivation to alter established habits. Public health initiatives targeting this demographic should thus balance health education with realistic, age-sensitive approaches that enhance enjoyment, independence, and social engagement in eating.

## Data Availability

The original contributions presented in the study are included in the article/supplementary material, further inquiries can be directed to the corresponding author.
